# A highly antigenic fragment within the zoonotic *Cryptosporidium parvum* Gp900 glycoprotein (Domain 3) is absent in human restricted *Cryptosporidium* species

**DOI:** 10.1371/journal.pone.0287997

**Published:** 2023-08-17

**Authors:** Denise Ann E. Dayao, Justyna J. Jaskiewicz, Abhineet S. Sheoran, Giovanni Widmer, Saul Tzipori

**Affiliations:** 1 Department of Infectious Disease and Global Health, Tufts University Cummings School of Veterinary Medicine, North Grafton, MA, United States of America; 2 BioMEMS Resource Center, Center for Engineering in Medicine and Surgery, Department of Surgery, Massachusetts General Hospital, Harvard Medical School, and Shriners Hospitals for Children, Boston, MA, United States of America; Foshan University, CHINA

## Abstract

We identified a fragment (Domain 3—D3) of the immunodominant sporozoite surface glycoprotein of the zoonotic parasite *Cryptosporidium* gp900, which is absent *C*. *hominis* and *C*. *parvum anthroponosum*. The fragment is highly antigenic and is able to effectively differentiate between zoonotic *C*. *parvum* and species/genotypes that infect preferentially humans. D3 detection provides a serological tool to determine whether the source of human cryptosporidiosis is of animal or human origin. We demonstrate this in experimentally challenged piglets, mice, rats, and alpaca. We speculate that the absence of this fragment from the *C*. *hominis* and *C*. *parvum anthroponosum* gp900 protein may play a key role in their host restriction.

## Introduction

Cryptosporidiosis is a serious cause of morbidity and mortality in children and contributes to acute and/or chronic diarrhea, growth retardation, malnutrition, cognitive impairment, or death [1, 2]. The Global Enteric Multicenter Study (GEMS) has found that *Cryptosporidium* ranked second only to rotavirus as a cause of moderate to severe diarrhea in children ≤ 2 years old [3–5]. Two *Cryptosporidium* species are primarily responsible for the human disease, namely *C*. *hominis* and *C*. *parvum*. Several studies from Africa have shown that approximately 75% of infected children excrete *C*. *hominis*, 20% excrete *C*. *parvum*, and 5% excrete a mixture and others [6, 7]. When *C*. *parvum* isolates collected during the GEM study were further analyzed, >95% of them were classified as anthroponotic (also known as *C*. *p*. subsp. *anthroponosum*), sharing many phenotypic and genetic characteristics with *C*. *hominis* [6] including restriction to humans. The zoonotic *C*. *parvum* (*C*. *parvum* subspecies *parvum* or *C*. *p*. *parvum*) and *C*. *hominis*, the two species infecting humans while genetically close, are only partially cross-protective [8, 9], which may impact the design of vaccines against human cryptosporidiosis.

The existence of named mammalian species within the genera *Cryptosporidium* have been known for several decades based entirely on host predilection and/or by their distinct genetic profile. Antigenic differences in the *p23* and *gp900* surface glycoprotein genes were first described by Sturbaum et al. [10]. The authors developed monoclonal antibodies against *gp23* and *gp900* selective for zoonotic *C*. *parvum* IOWA to screen solubilized glycoproteins from *C*. *hominis* isolates for reactivity. Our work explored a fragment of *gp900* that differentiates between zoonotic and anthroponotic *Cryptrosporidium* species/genotypes.

In this communication, we describe an immunogenic region, Domain 3 (D3), which resides within the surface glycoprotein gp900 [11] of *C*. *p*. *parvum* but is absent from *C*. *hominis* and anthroponotic *C*. *parvum (C*. *p*. *anthroponosum)*, both of which are human-restricted. We show that the antibody against D3 is expressed in the sera of all experimental animal species that we challenged or immunized with zoonotic *C*. *p*. *parvum*, but in none of those challenged or immunized with *C*. *hominis* and *C*. *p*. *anthroponosum*. In addition, a survey of the genome of *C*. *hominis*, *C*. *parvum* and other *Cryptosporidium* species is consistent with our observations in showing the presence of gp900 D3 in zoonotic species and the absence in anthroponotic species.

## Materials and methods

### Ethics statement

All experiments were performed in strict accordance with the recommendations set forth by the National Institutes of Health Guide for the Care and Use of Laboratory Animals (8th Edition). Protocols were approved by the Institutional Animal Care and Use Committee at Tufts University Cummings School of Veterinary Medicine (Animal Welfare Assurance Number D16-00572 [A4059-01]). Every effort was made to minimize animal suffering and distress over the course of studies performed. Mice and rats were euthanized by CO_2_ asphyxiation, followed by cervical dislocation, while GB piglets were euthanized by intracardiac injection of phenytoin and pentobarbital (Somnasol, 100 mg/kg) following intramuscular injection of ketamine (100 mg/kg) and xylazine (5 mg/kg).

### Parasites

The *C*. *hominis* strain TU502 and the *C*. *p*. *anthroponosum* isolate TU114 were originally isolated from children in Uganda [12, 13]. TU502 is maintained and serially passaged in gnotobiotic (GB) piglets, while TU114 is propagated in immuno-suppressed mice or GB piglets at Tufts University. Oocysts were purified from gut contents and feces on Nycodenz step gradients (Alere Technologies AS, Norway) as described previously [14]. The zoonotic *C*. *p*. *parvum* IOWA strain was obtained from Bunchgrass Farm (Deary, ID, USA). Before inoculation or use for antigen preparation, oocysts were treated with 10% bleach (0.5% sodium hypochlorite) for 7 min on ice to eliminate bacterial contaminants, followed by three washes with distilled water and centrifugation at 18,000 g for 2 min.

### Antigen/Protein preparation

*Cryptosporidium* antigens containing proteins used for immunizations and ELISAs, were prepared as described previously [15]. Briefly, oocysts were bleached and washed with PBS as described above and were then excysted in 1.5% taurocholic acid in sterile PBS for 1 h at 37°C. Supernatant containing shed antigens was collected after centrifugation (18,000 xg, 2 min). The pelleted fraction was additionally processed by 7 cycles of freeze and thaw and then combined with the supernatant. Antigens were stored in -20°C until use. For rat immunizations, antigens from 1x10^6^ oocysts from *C*. *hominis* TU502, *C*. *p*. *anthroponosum* TU114 or *C*. *p*. *parvum* IOWA were used for each animal. For mouse immunizations, oocyst lysates were used as described previously [16].

A synthetic gene was prepared encoding the entire 159 aa D3 sequence from *Cp*- *gp900* aa638-796 (gene cgd7_4020 cryptodb.org). The coding DNA was inserted into a pET32 vector and expressed in fusion with *E*. *coli* thioredoxin and a carboxyl terminal end myc epitope. The protein was expressed in *E*. *coli*, purified by nickel affinity and quantified as described for other proteins [15].

### Animals and animal procedures

Female BALB/c mice or Sprague Dawley rats (Charles River Laboratories, USA) were housed in bedded cages and randomly assigned groups with access to food and water ad libitum. Mice were infected orally using a pipette tip with freshly bleached and washed *Cryptosporidium* oocysts in 20 μl sterile distilled water. GB piglets, delivered via Cesarean section, were maintained inside sterile isolators, and were fed three times daily with a total of 500–700 ml/day of human infant milk replacer (Similac Advance Ready to Feed Infant Formula, Abbott Nutrition). Piglets were infected with oocysts in 2 ml sterile saline via gastric gavage using a stainless inoculating needle. Blood samples were collected from the facial (mice), jugular (alpaca) veins and heart (piglets and rats). Serum was separated from the whole blood by centrifugation at 1,800 g for 10 minutes. Fresh fecal pellets were collected while manually restraining each mouse. Fecal pellets were suspended in 5 volumes of saline with protease inhibitor (Sigma catalog number P2714), homogenized, and centrifuged at 18,000 g for 3 min. Supernatant was collected and stored at -20°C. Saliva was collected by inserting a piece of filter paper (6 millimeter in diameter) using forceps into the mouth of each mouse and saturating the filter paper with mouse saliva. Filter paper with saliva from individual mice was suspended in 100 μl saline with protease inhibitor and stored at -20°C.

### Animal experiments

#### Alpaca

A 7.5-years old male alpaca (*Vicugna pacos*) was immunized with *C*. *hominis* TU502 antigen. The immunization was performed seven times to achieve enhanced antibody affinity maturation and obtain high titer antibodies, at 2-4-week intervals. For each immunization, 1.525 mg antigen was mixed with CpG (Innaxon, Adipogen Life Technologies) and alum (Alhydrogel, Invivogen) adjuvants. Antigen and adjuvant mixture (5 ml) was admistered as multiple subcutaneous injections under the skin of the lateral chest area or just behind the shoulder. Two weeks after the last immunization, whole blood was collected from the jugular vein of the alpaca.

#### Rats

One or two female Sprague Dawley rats, 3–4 weeks of age, were immunized with antigen from *C*. *hominis* TU502, *C*. *p*. *parvum* IOWA, *C*. *p*. *anthroponosum* TU114 or gp900 D3 proteins. A single immunization contained the 1x10^6^ excysted oocysts antigen or 50 μg gp900 D3 protein suspended in sterile saline and adjuvant (CpG and Alhydrogel or Freund’s complete or incomplete). A control rat which received sterile saline and adjuvant was included in the experiment. Rats were immunized (200 μl, in two separate intramuscular injections) on day 0, 35 and 56. Three weeks after the last boost, blood was collected from each rat at euthanasia. Serum samples were tested for *Cryptosporidium* (*C*. *hominis*, *C*. *p*. *parvum* or gp900 D3) specific IgG by ELISA. Sera were also used in cell infection assay to determine if immunized sera can protect from zoonotic *C*. *p*. *parvum* infection *in vitro*.

#### GB piglets

Piglet (*Sus scrofa domesticus*) sera tested for *Cryptosporidium*-specific IgG were generated from our previous experiments [9]. Briefly, 3-5-day old GB piglets were orally infected (2 ml) with 1 x 10^4^ *C*. *p*. *anthroponosum* TU114 oocysts (primary infection) and then following recovery (~33–45 days after birth) again with *C*. *p*. *anthroponosum* TU114 (n = 4) or *C*. *hominis* TU502 (n = 8) (secondary infection). Piglets were considered recovered from primary infection when oocyst excretion and PCR were negative for ≥3 consecutive days. Age matched controls did not receive any infection at the time of the primary infection and received *C*. *p*. *anthroponosum* TU114 (n = 3) or *C*. *hominis* TU502 (n = 4) at the time of secondary infection. Ten days after the secondary infection, piglets were bled and serum samples were collected. *Cryptosporidium*-specific IgG was determined in each serum sample by ELISA.

### Mice

Experiment 1. Serum samples were obtained from our previously reported mouse experiment [16], which involved intraperitoneal (i. p.) immunization with *C*. *p*. *parvum* IOWA or with *C*. *hominis* oocyst lysates. Briefly, four groups of 3- to 4-week-old BALB/c mice, 4 to 5 mice per group, were assigned as follows: five mice were administered with lysate of 1x10^6^ *C*. *hominis* oocysts; another group of 5 mice were administered with lysate of 1x10^6^ *C*. *p*. *parvum* IOWA oocysts; controls (n = 5) were orally infected with 1 x 10^6^ *C*. *p*. *parvum* IOWA oocysts; four mice were unimmunized controls. Mice immunized i.p. (100 μl) with oocyst lysates three times at 2-to 3-week intervals. The control mice were administered orally (20 μl) with oocysts once same time as the first immunization of the experimental groups. Blood samples were collected from individual mice 2 weeks after the last immunization.

Experiment 2 involved 10, 3–4-week-old BALB/c mice. Five mice were immunized with gp900 D3 protein expressed in *E*. *coli* and the other five, which served as controls, received sterile saline. Mice were immunized on day 0, 35 and 56. A single immunization (100 μl in two separate intramuscular injections) contained 20 μg gp900 D3 protein suspended in sterile saline and Freund’s complete or incomplete adjuvant (Sigma, USA). Three weeks after the last immunization, blood, feces and saliva were collected for antibody detection from each mouse prior to challenge. All mice were pretreated with i.p. injections of IFN-y-neutralizing MAb two hours before oral challenge with 1x10^6^ zoonotic *C*. *p*. *parvum* IOWA oocysts. Oocyst shedding of individual mice was quantified by quantitative PCR (qPCR) as previously described [17].

#### Serology

Blood samples were collected from each animal after immunization or challenge to test for a *Cryptosporidium-*specific (*C*. *p*. *parvum* IOWA, *C*. *hominis* TU502 or gp900 D3) IgG response by enzyme linked immunosorbent assay (ELISAs). *Cryptosporidium*- specific IgG was monitored in serum, respectively, using a previously described ELISA protocol [16]. *Cryptosporidium*-specific IgG bound to *Cryptosporidium* antigens or gp900 D3 were monitored with a horseradish peroxidase anti-mouse IgG (Southern Biotechnology catalog number 1030–05), anti-pig IgG (Bethyl Laboratories catalog number A100-104-P), anti-llama IgG (Bethyl Laboratories catalog number A160-100P) and anti-rat IgG (Southern Biotech catalog number 3051–05).

#### *Cryptosporidium*- specific IgA determination in feces and saliva

In mouse experiment #2, *Cryptosporidium*-specific IgA was determined in feces and saliva of mice. Feces and saliva were collected from each mouse after immunization or challenge. *Cryptosporidium*- specific IgA was detected in the supernatant from fecal sample homogenates and saliva by ELISA. IgA bound to *Cryptosporidium* antigens or gp900 D3 was measured with a horseradish peroxidase anti-mouse IgA (Invitrogen, Catalog number 62–6720).

### Cell infection assay

The effect of anti-*C*. *p*. *parvum* antibodies raised in serum of immunized rats was examined using in vitro infection assay as previously described [15] with few modifications. Briefly, 2x10^4^ Madin-Darby bovine kidney (MDBK) cells (ATCC: CCL-22) were grown into each well of a 96-well culture plate. Cells were incubated at 37°C with 5% CO_2_ in RPMI media supplemented with 10% fetal bovine serum, 100 IU/ml of penicillin/50 mg/ml of streptomycin sulfate solution for 24 hours until confluent (approximately 4x10^4^ cells/well). To stimulate excystation, pre-bleached *C*. *p*. *parvum* IOWA oocysts (calculated to have 2.5 x 10^4^ per well) were incubated in 1.5% taurocholic acid in sterile saline at 37°C for 30 min. Pre-excysted oocysts were incubated with different dilutions (1:100, 1:200, 1:400 and 1:800) of post immune rat sera at 37°C for 30 minutes. Sporozoites and serum solution were then added onto confluent cell monolayers in the presence of uninfected controls and incubated for 24 hours at 37°C in CO_2_ incubator. Incubation was terminated 24 hours post infection and stained for immunofluorescent microscopy analysis as described by Jaskiewicz, et al., 2021 [15].

### DNA extraction and qPCR

Genomic DNA was extracted from freshly collected feces from individual mice using the E.Z.N.A. Stool DNA Kit (Omega, Biotek catalog number D4015-02). qPCR was based on detection of copies of *Cryptosporidium* heat shock protein gene (HSP70) [17] and completed using a Step-One Plus instrument (Applied Biosystems, USA).

### Statistical analysis

All data were analyzed using GraphPad Prism version 9.4.0. The Mann-Whitney t-test, Kruskal-Wallis, ANOVA or 2-way ANOVA with Dunn’s or Dunnett’s multiple-comparisons test were used to compare groups, as indicated in the figure legends. All results are expressed as means standard deviations (SD). P values of ≤0.05 were considered statistically significant (*, P<0.05; **, P< 0.01, ***, P< 0.001).

## Results

### Animal experiments

#### Alpaca

**Fig 1:**
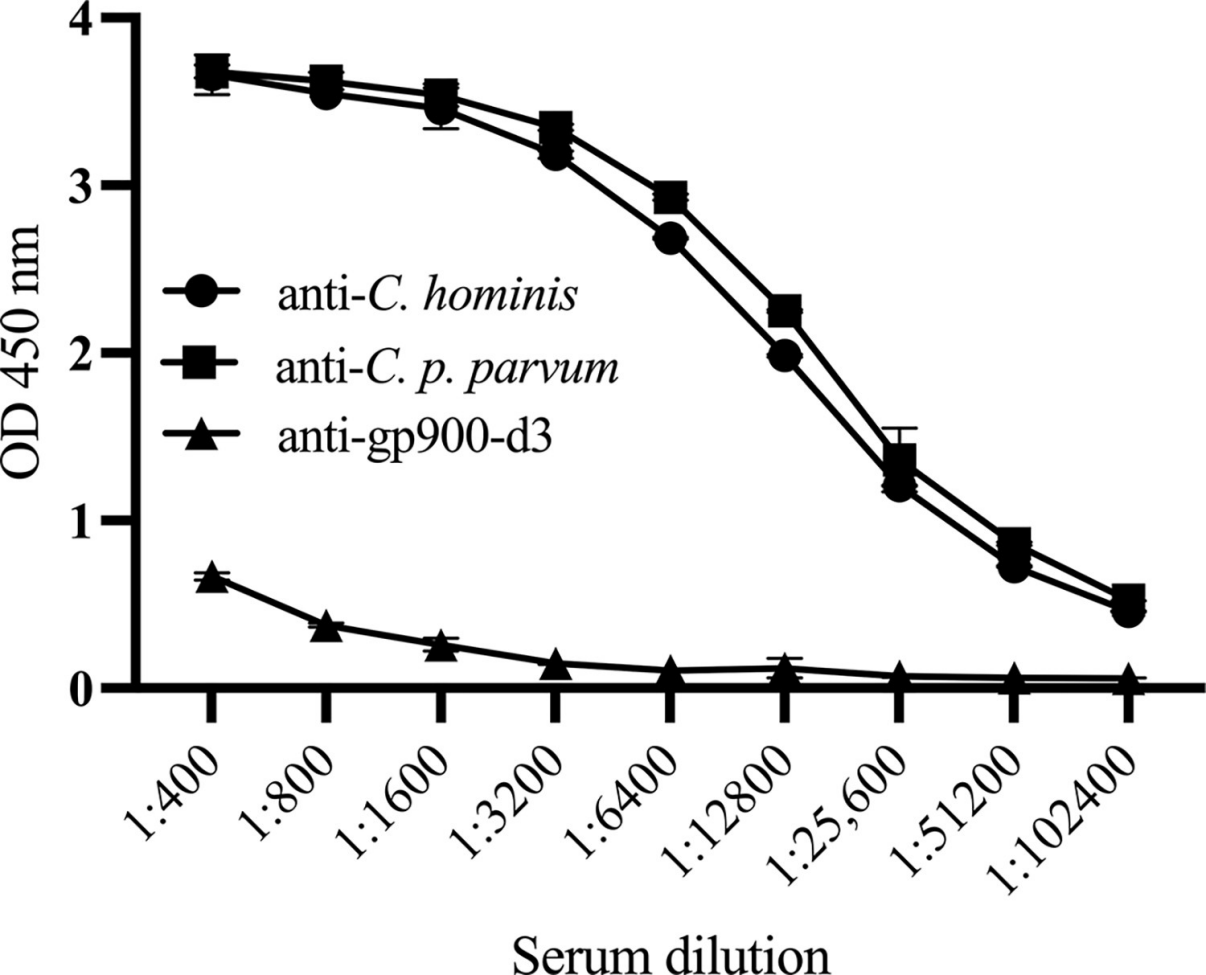
*Cryptosporidium*-specific IgG in serum of *C*. *hominis* TU502 immunized alpaca. Each data point is the mean OD 450nm of duplicates of specified serum dilutions obtained when tested against *Cryptosporidium* antigens (*C*. *hominis*, *C*. *p*. *parvum* IOWA and gp900 D3). An alpaca was immunized with *C*. *hominis* TU502 oocyst lysates subcutaneously with CpG and Alhydrogel adjuvants, 7 times at 2–4 weeks intervals. Two weeks after the last immunization serum was collected and the anti-*C*. *hominis* TU502, *C*. *p*. *parvum* IOWA and gp900 D3 IgG was determined by ELISA. Statistical comparisons: anti-*C*. *hominis* TU502 vs. anti-gp900-d3, p = 0.0021; anti-zoonotic *C*. *p*. *parvum* IOWA vs. anti-gp900-d3, p = 0.0008, Kruskal-Wallis test with Dunn’s multiple comparison test.

The immune serum collected from an alpaca immunized with *C*. *hominis* TU502 antigen was tested for *C*. *hominis*, *C*. *p*. *parvum* IOWA and gp900 D3 specific IgG. The results of the ELISAs are shown in **Fig 1**. The immune serum reacted strongly against *C*. *hominis* and *C*. *p*. *parvum* IOWA antigens with ≥1:102,400 titer for both antigens. In contrast, the reaction against gp900 D3 was weaker (anti-*C*. *hominis* TU502 vs. anti-gp900-d3, p = 0.0021; anti-zoonotic *C*. *p*. *parvum* IOWA vs. anti-gp900-d3, p = 0.0008, Kruskal-Wallis test with Dunn’s multiple comparison test).

#### Rats

**Fig 2:**
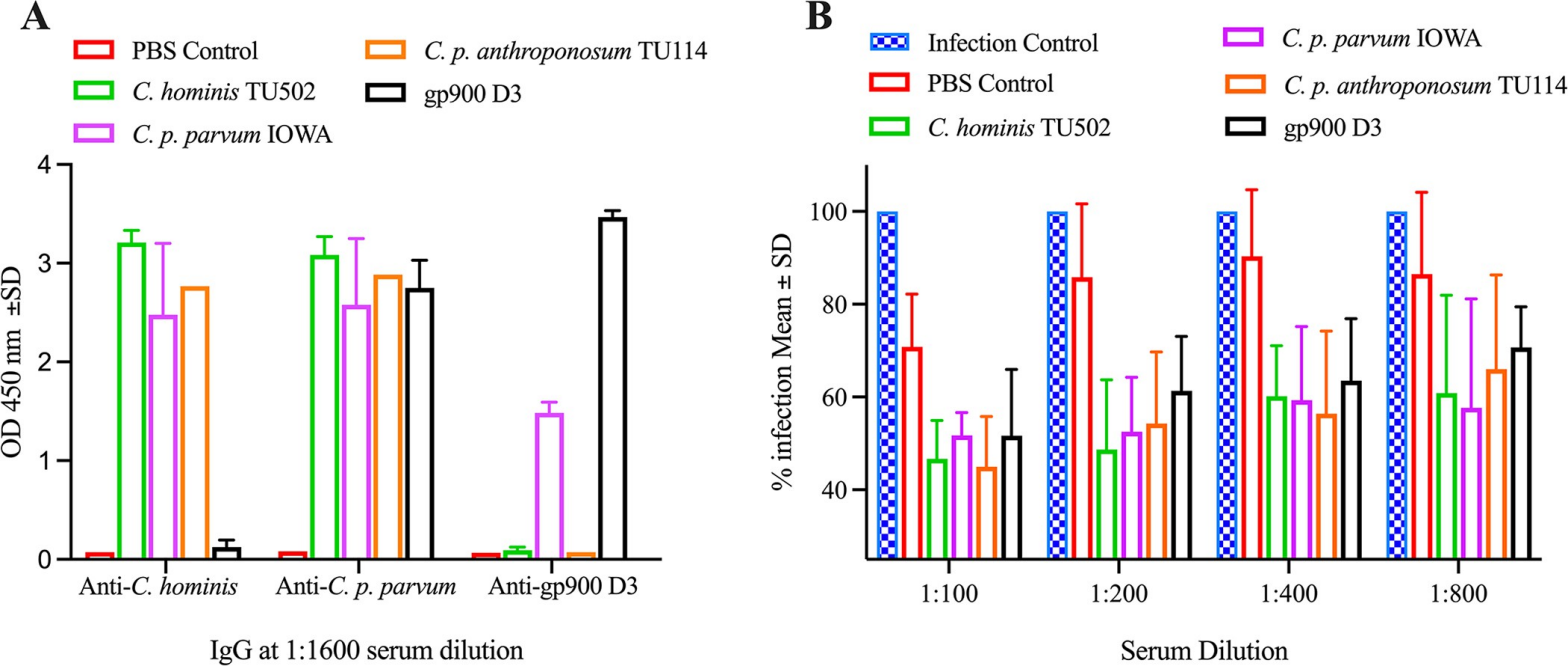
*Cryptosporidium*-specific IgG in post immune rat sera and the effect of these sera in *C*. *parvum* infection in vitro. Rats were immunized 3 times on day 0, 35 and 56 with sporozoite lysates of *C*. *hominis* TU502 (n = 2), zoonotic *C*. *parvum* (n = 2) or *C*. *p*. *anthroponosum* TU114 (n = 1), with each immunization containing lysate of 10^6^ excysted oocysts. Additional rats (n = 2) were immunized with or *C*. *p*. *parvum* derived gp900 D3 or PBS as control (n = 1). **(A)** shows the high level of *Cryptosporidium*-specific IgG (anti-*C*. *hominis*, anti-*C*. *p*. *parvum* IOWA and anti-gp900 D3) in post-immune sera in comparison to serum from the control rat. Values indicate optical densities (OD) measured at 450-nm absorbance of the average of technical duplicates at 1:1600 serum dilution. Post immune *C*. *hominis*, *C*. *p*. *anthroponosum* TU114 and control had low level of gp900 domain 3 IgG. **(B)** The effect of post-immune sera to inhibit intracellular *C*. *parvum* infection *in vitro* (using MDBK cells) was determined by adding sera at 1:100, 1:200, 1:400 and 1:800 dilutions. Infection control was a *C*. *p*. *parvum* IOWA cell culture maintained in growth medium alone. Rate of infection (%) in the presence of sera were normalized using data from infection control. Comparison of infection rate between cultures with post immune and age-matched PBS control was analyzed using 2-way ANOVA with Dunnett’s multiple comparisons test. Data shown are mean ±SD from duplicate assays.

To determine if *Cryptosporidium* immunized rats had antibody against *C*. *p*. *parvum*, *C*. *hominis* and gp900 D3, post immune sera were tested for specific IgG response. The outcome of rat immunizations with the three parasite antigens are summarized in **Table 1 and Fig 2A**. The serum from the control rat did not react to any of the three antigens. *C*. *p*. *parvum* IOWA strongly responded against all three antigens whereas gp900 post immune sera reacted to *C*. *p*. *parvum* IOWA and gp900 D3 antigens only. *C*. *hominis* TU502 and *C*. *p*. *anthroponosum* TU114 immune sera had high anti- *C*. *hominis* TU502 and *C*. *p*. *parvum* IOWA and low level of gp900 domain 3 IgG.

**Table 1:** Rat immunization regimen and their serum antibody response against *Cryptosporidium* antigens determined by ELISA^a^.

Number of rats	Immunized with	Dose for each immunization	Antigen		
			*C*.* hominis* TU502	*C*.* p*.* parvum* IOWA	gp900 D3
2	*C*. *hominis* TU502	10^6^ oocysts^c^	++	++	-
2	*C*. *p*. *parvum* IOWA	10^6^ oocysts^c^	++	++	+
1	*C*.* p*. *anthroponosum* TU114	10^6^ oocysts^c^	++	++	-
2	gp900 D3^b^	50 μg	-	++	++
1	PBS (control)	n/a	-	-	-

^a^Rats were immunized intramuscularly three times with antigens indicated above on day 0, 35 and 56. Three weeks after the last immunization, sera (1:1600 dilution) from rats were tested for *Cryptosporidium*-specific IgG antibody.

^b^Recombinant proteins expressed in *E*. *coli* or insect cells.

^c^Rats were immunized with materials from post-excystation of the above number of oocysts, prepared as described in the matherials and methods; ++, strong reaction; +, moderate reaction-, no reaction

The effect of *C*. *p*. *parvum* antiserum on *C*. *p*. *parvum* invasion of cultured cells was evaluated using MDBK cells (**Fig 2B**). *C*. *p*. *parvum* IOWA pre-excysted oocysts were added to 1:100, 1:200, 1:400 and 1:800 dilutions of each post immune serum, age matched PBS control rat serum or media alone as negative control. The rate of infection (%) was normalized against the medium control. When compared to level of infection obtained in cultures treated with age-matched PBS control rat serum, 1:100, 1:200, 1:400 and 1:800 serum dilutions from *C*. *p*. *parvum* IOWA (p = 0.0311, <0.0001, 0.0001 and 0.0004, respectively), *C*. *hominis* TU502 (0.004, <0.0001, 0.0002 and 0.002, for respective dilutions) and *C*. *p*. *anthroponosum* TU114 (p = 0.061, 0.0006, 0.0002 and 0.0393, respectively) infected rats significantly reduced parasite invasion in vitro. Post-immune serum from rats immunized with gp900 D3 reduced the infection level at 1:100, 1:200 and 1:400 dilutions (p = 0.0301, 0.0034; and 0.0012, respectively). The comparison of the infection level between cultures supplemented with post-immune serum and age-matched PBS control was analyzed using 2-way ANOVA with Dunnett’s multiple comparisons test.

### GB piglets

**Fig 3:**
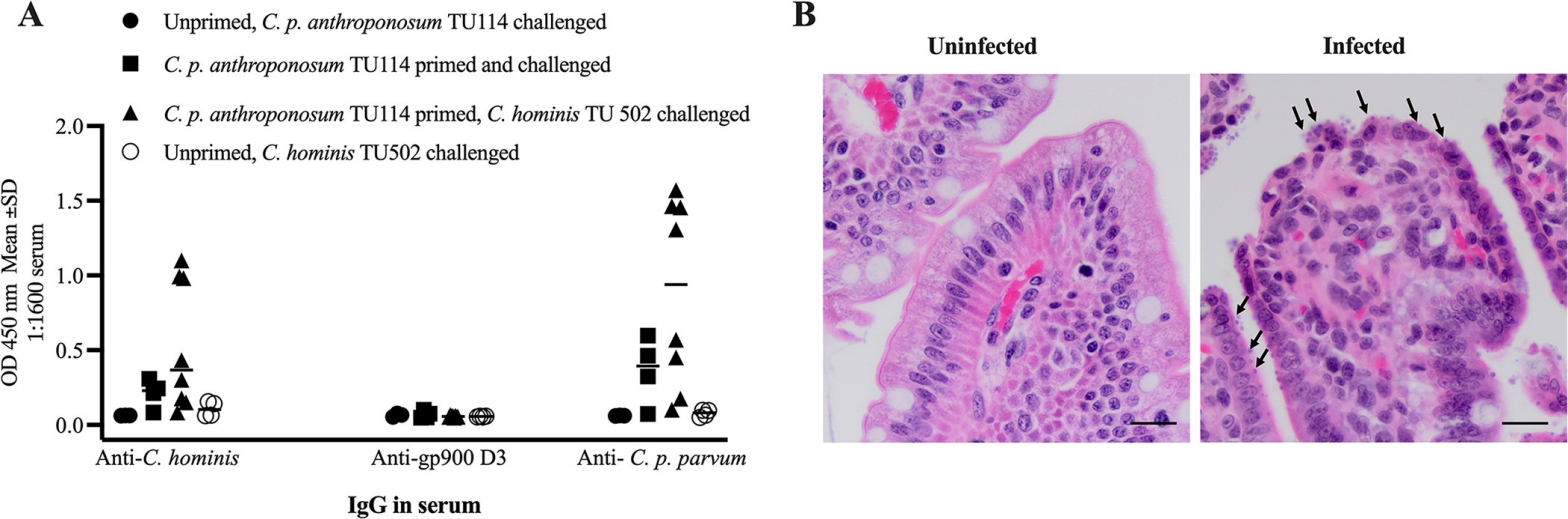
*Cryptosporidium*-specific IgG in gnotobiotic post-immune sera and *C*. *hominis* infection in piglet gut. 3–5-day old gnotobiotic piglets received a primary infection with *C*. *p*. *anthroponosum* TU114 (n = 4) or *C*. *hominis* TU502 (n = 8) or remained uninfected (unprimed) and were challenged (secondary infection) with *C*. *p*. *anthroponosum* TU114 (n = 3) or *C*. *hominis* TU502 (n = 4) after recovery (~33–45 days after birth). (A) *Cryptosporidium*-specific IgG was determined from sera collected 10 days post-challenge. Some sera from primed and challenged piglets had elevated anti- *C*. *p*. *parvum* or *C*. *hominis* in contrast to undetectable anti-gp900 D3 response in all sera regardless of infections received (unprimed/primed). Each data point is the mean OD 450nm of duplicates at 1:1600 serum dilution obtained when tested against *Cryptosporidium* antigens. (B) Sections of terminal ilea of gnotobiotic piglets. The left image shows a normal villi coated with normal enterocytes. The right image shows the heavily infected enterocytes with *C*. *hominis* forms (arrows) embedded in the microvillus border. The tip of the infected section is covered by irregular and damaged layer of enterocytes (H & E; M– 400x). Scale indicates 20μm.

The outcome of the experiments in GB piglets, orally infected with 1 x 10^4^ *C*. *p*. *anthroponosum* TU114 oocysts (primary infection) and reinfected with *C*. *p*. *anthroponosum* TU114 or *C*. *hominis* TU502 (secondary infection/challenge) are shown in **Table 2 and Fig 3A**. *Cryptosporidium*-specific IgG was determined in serum collected 10 days post-challenge. Some piglet sera from primed and challenged piglets had elevated anti-*C*. *p*. *parvum* IOWA or *C*. *hominis* TU502 antibody compared to undetectable anti-gp900 D3 response in all sera regardless of the infections received (unprimed/primed). Unprimed piglets whether challenged with *C*. *p*. *anthroponosum* TU114 *C*. *hominis* TU502 or did not show reaction to any of the three antigens. These piglets were sampled 10 days post-challenge hence were not able to produce detectable *Cryptosporidium* specific IgG. Unprimed and challenged piglets heavily shed oocysts in feces after challenge. Histopathologic section stained with haematoxylin and eosin (H&E, Fig 3B) shows the terminal ileum of a GB piglet with *C*. *hominis* robust infection.

**Table 2:** Immune responses against *Cryptosporidium*-specific antigens in sera of gnotobiotic piglets orally infected with anthroponotic *Cryptosporidium* spp. oocysts.

Number of piglets	Primary/ secondary infection and dose	Response against		
		*C*.* hominis* TU502	*C*.* p*.* parvum* IOWA	gp900 D3
3	None/ 10^7^ *C*.* p*. *anthroponosum* TU114	-	-	-
4	None/ 10^7^ *C*. *hominis* TU502	-	-	-
8	10^4^ *C*.* p*. *anthroponosum* TU114/	++	++	-
	10^7^ *C*.* hominis* TU502			
4	10^4^ *C*.* p*. *anthroponosum* TU114/ 10^7 ^*C*.* p*. *anthroponosum* TU114	+	+	-

Sera were tested at 1:1600 dilution by ELISA, ++, strong reaction; +, moderate reaction-, no reaction

### Mice

**Fig 4:**
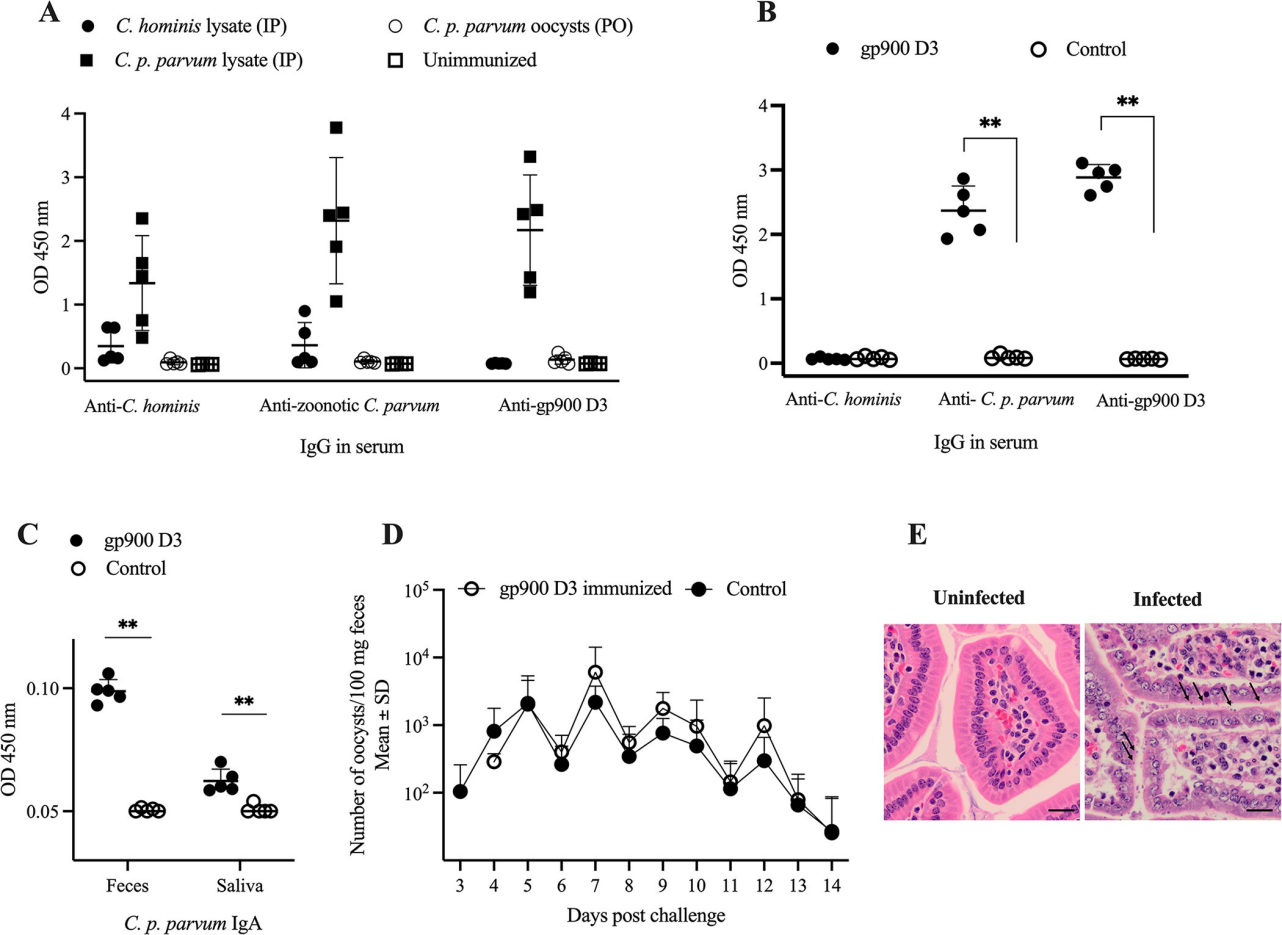
Reaction of mouse post-immune sera against *Cryptosporidium* antigens and the effect of gp900 domain 3 immunization on parasite shedding after challenge. (A) Mice were immunized 3 times with *C*. *hominis* TU502 (n = 5) or zoonotic *C*. *p*. *parvum* (n = 5) lysates intraperitoneally (IP), or once with zoonotic *C*. *p*. *parvum* oocysts (n = 5) orally (PO). Four unimmunized mice served as controls. *C*. *p*. *parvum* lysate immune sera had high IgG level against *C*. *p*. *parvum* and gp900 D3 and lower anti-*C*. *hominis* IgG level (Kruskal-Wallis with Dunn’s multiple comparisons test: anti- *C*. *p*. *parvum* vs. anti-gp900 domain 3, p = >0.9999; anti- *C*. *p*. *parvum* IgG vs. anti-*C*. *hominis*, p = 0.0267; and anti-gp900 domain 3 vs. anti-*C*. *hominis*, p = 0.0216). (B) 3–4-week-old BALB/c mice (n = 5) were immunized with recombinant gp900 D3 protein or PBS (controls, n = 5) on day 0, 35 and 56. *Cryptosporidium* specific antibodies were measured three weeks after the last immunization. Immunized mice had elevated level of anti-*C*. *p*. *parvum* and anti-gp900 D3 specific IgG compared to controls (Mann Whitney test, p = 0.008 and 0.0079, respectively), while anti-*C*. *hominis* IgG was undetectable. (C) gp900 D3 immunized mice had higher anti- *C*. *p*. *parvum* IgA in feces and saliva when compared to unimmunized controls (Mann Whitney test, p = 0.0079 and 0.0097, respectively). (D) gp900 domain 3 immunized and unimmunized control mice were challenged with 10^6^ *C*. *p*. *parvum* oocysts. A quantitative PCR showed a minor difference where oocyst excretion in the systemically D3 immunized mice was delayed by 24 hours as compared with the control. Error bars show SD. (E) Sections of terminal ilea of mice: left image (uninfected mouse) shows a normal villi coated with normal enterocytes; the right image (mouse with cryptosporidiosis) shows the moderately infected enterocytes embedded in the microvillus border (arrows). The infected section is covered by normal healthy layer of enterocytes. (H & E; M– 400x). Scale indicates 20μm.

The outcome of mouse experiment 1, which aimed to determine if the serum from mice immunized with *C*. *p*. *parvum* or *C*. *hominis* oocyst lysates systemically (i.p.) or *C*. *p*. *parvum* oocyts given orally recognize *C*. *hominis* TU502, *C*. *p*. *parvum* or gp900 D3 antigens, are presented in **Fig 4A**. Sera were tested at 1:1600 dilution. All unimmunized and mice immunized with *C*. *hominis* oocyst lysate and *C*. *p*. *parvum* oocysts did not generate serum IgG. *C*. *p*. *parvum* lysate immune sera had high IgG level against *C*. *p*. *parvum* and gp900 D3 and lower anti-*C*. *hominis* IgG level (Kruskal-Wallis with Dunn’s multiple comparisons test: anti *C*. *p*. *parvum* vs. anti-gp900 domain 3, p = >0.9999; anti *C*. *p*. *parvum* IgG vs. anti-*C*. *hominis*, p = 0.0267; and anti-gp900 domain 3 vs. anti-*C*. *hominis*, p = 0.0216).

The outcome of mouse experiment 2 which was intended to determine whether gp900 D3 protein is immunogenic and protect mice against challenge with *C*. *p*. *parvum* is presented in **Fig 4B–4D**. Five mice received three systemic immunizations with gp900 D3 while five control mice received PBS only. Three weeks after the last boost, *Cryptosporidium* specific antibodies were determined in feces, saliva and sera. Sera from gp900 D3 immunized mice had elevated anti-*C*. *p*. *parvum* and anti-gp900 D3 specific IgG in contrast to sera from controls (Mann Whitney test, p = 0.008 and 0.0079, respectively), while anti-*C*. *hominis* IgG was undetectable (**Fig 4B**). gp900 D3 immunized mice had elevated anti- *C*. *p*. *parvum* IgA in feces and saliva compared to unimmunized controls (Mann Whitney test, p = 0.0079 and 0.0097, respectively) (**Fig 4C**). To determine whether gp900 D3 immunizations give protection against *C*. *p*. *parvum* challenge, three weeks after the last immunization, mice were treated i.p. with 1 mg of IFN-γ -neutralizing MAb 2 hours prior to oral challenge with 10^6^ *C*. *p*. *parvum* oocysts. As determined by qPCR, control mice started to shed *Cryptosporidium* in feces on day 3 while immunized mice started on day 4 post-challenge. However, no significant difference was found between oocyst output in immunized and that of control mice (**Fig 4D**). Histopathologic section of the terminal ileum of a mouse with cryptosporidiosis showed moderate parasite infection of the enterocytes (**Fig 4E**).

## Discussion

The study of *Cryptosporidium* populations and transmission cycles has relied extensively on genetic polymorphisms. Insight from this research has largely confirmed the existence of host-species adapted *Cryptosporidium* populations, which in many cases has been elevated to species [18–22]. Because of extensive DNA sequence polymorphism, two abundant *Cryptosporidium* sporozoite surface glycoproteins, gp900 and gp60 [23], have attracted much research interest. Not only has the large number of alleles detected in the species *C*. *parvum* and *C*. *hominis* [24] been extensively applied to understand the evolution of these species and the epidemiology of cryptosporidiosis, but the potential role of these proteins in the interaction with the host has also been highlighted [10, 25].

In contrast to extensive research aimed at exploring sequence divergence between *Cryptosporidium* genomes [26–28], antigenic differences among *Cryptosporidium* species and *C*. *parvum* subspecies have rarely been studied. Here we show that the presence of a highly antigenic fragment within the surface glycoprotein gp900 segregates to some extent with host range, as this domain is absent from *C*. *hominis* and *C*. *p*. *anthroponosum*, both of which are mostly parasites of humans. In contrast to epidemiological surveys using other recombinant *C*. *parvum* antigens without known polymorphism in *Cryptosporidium* species and *C*. *parvum* subspecies, here we focused on a relatively large antigenic domain which segregates with host specificity. Consistent with sequence analysis, experiments in alpacas, mice, GB piglets and rats demonstrate the specificity of the antibody response as recombinant D3 protein is only recognized in animals immunized or infected/challenged with a *Cryptosporidium* isolate predicted to encode gp900 D3. Monoclonal antibodies specific for *C*. *p*. *parvum* were previously identified [10] but this earlier work did not include *C*. *p*. *anthroponosum* isolates. In their analysis, Sturbaum and colleagues [10] speculate on the significance of sequence polymorphisms in gp900 and its relevance to host specificity. The identification of the *C*. *p*. *anthroponosum* lineage within *C*. *parvum* which shares a restricted host range with *C*. *hominis* raises interesting evolutionary questions. The extent to which gp900 determines or influences host range remains to be determined. In this context, extensive telomeric polymorphism between *C*. *p*. *parvum* and *C*. *p*. *anthroponosum* has also been noted and viewed as a potential location of host range associated loci [12, 27]. Both species consist of several subtypes that are linked to geographic distributions and host preferences, however the evolutionary adaptation processes to humans remains unclear. Nader and colleagues [27], analyzing some 21 whole-genome sequences to elucidate the evolution of anthroponomics, have shown that *C*. *parvum* splits into two subclades and that genetic exchange between *Cryptosporidium* subtypes plays a prominent role throughout the evolution of the genus. Analysis of 467 gp60 sequences collected from locations across the world shows that the population genetic structure differs markedly between the main zoonotic and the anthroponotic subtypes.

We investigated the D3 polymorphism in the context of other *Cryptosporidium* genome sequences. Nine complete gp900 amino acid sequences retrieved from GenBank included 3 versions from the zoonotic *C*. *parvum* IOWA, one *C*. *tyzzeri* [29], 2 *C*. *hominis* which includes the reference isolate TU502 [30] and one each of *C*. *parvum* chipmunk isolate, *C*. *bovis* and *C*. *felis*. In these sequences, only *C*. *parvum* and *C*. *tyzzeri* gp900 possess D3, while this domain is absent from the other sequences. When searching GenBank with the D3 amino acid sequence only, significantly similar sequences are found in other *Cryptosporidium* species. The similarities of D3 with non-*parvum* sequences are lower though, but they are still highly significant. Thus, it appears that D3-like motifs are present in other *Cryptosporidium* genomes, perhaps located in different genes. There could certainly be errors in the complete genomes we have used, because the long threonine repeats may have introduced errors during genome assembly.

While our experiments suggest a link between the absence of this highly antigenic D3 and host restriction of *Cryptosporidium* found in humans, this is not a whole or non-phenomenon, as we were able to experimentally infect calves with high doses of *C*. *hominis* TU502 and with *C*.*p*. *anthroponosum* TU114. The infections however were asymptomatic resulting in a minimal oocyst excretion on day 10 after infection as compared with zoonotic *C*. *p*. *parvum* infections (unpublished data). This suggests that it is not due to lack of specific cell receptors in calves but, perhaps, the presence of D3 enhanced infections in calves. Furthermore, we have shown that rats, normally resistant to *C*. *hominis* infection, can be infected intratracheally with *C*. *hominis* [31]. Also to be considered is the possibility of mis-assembled genomic contigs. Errors in complete genome sequences could have been caused by long threonine repeats flanking the D3 domain.

Despite a substantial level of D3 specific serum IgG in mice systemically immunized with D3 protein, it only delayed oocyst excretion by one day as comparetd with unimmunized control. It is however possible that direct immunization of the gut mucosa with D3 protein might have limited more substantially the degree of infectivity of the zoonotic *C*. *p*. *parvum* as compared with unimmunized control. Unfortunatley delivery of protein directly to the gut mucosa remains challenging.

This communication reports the first *Cryptosporidium* antigen capable of distinguishing between the human-restricted *C*. *hominis* and *C*. *p*. *anthroponosum* and the *C*. *p*. *parvum* which infect most mammals causing serious disease in humans and in young ruminants. We speculate that D3 presence in zoonotic *C*. *parvum* isolates may enhance their ability to infect many if not all mammalian species. Conversely, the lack of D3 may be a key to limiting their host range. These observations regarding D3 may provide a serological tool to determine whether the source of human cryptosporidiosis is of animal or human origin.
